# Supplementary material for: DR3 stimulation of adipose resident ILC2s ameliorates type 2 diabetes mellitus
Source: Nat Commun. 2020 Sep 18;11:4718. doi: 10.1038/s41467-020-18601-7 (PMC7501856; doi:10.1038/s41467-020-18601-7)
Supplement: Supplementary file 1 — Supplementary Information [file 41467_2020_18601_MOESM1_ESM.pdf]

DR3 stimulation of adipose resident ILC2s ameliorates type 2 diabetes mellitus

Shafiei-Jahani et. al, Supplementary information.

Supplementary Figure 1

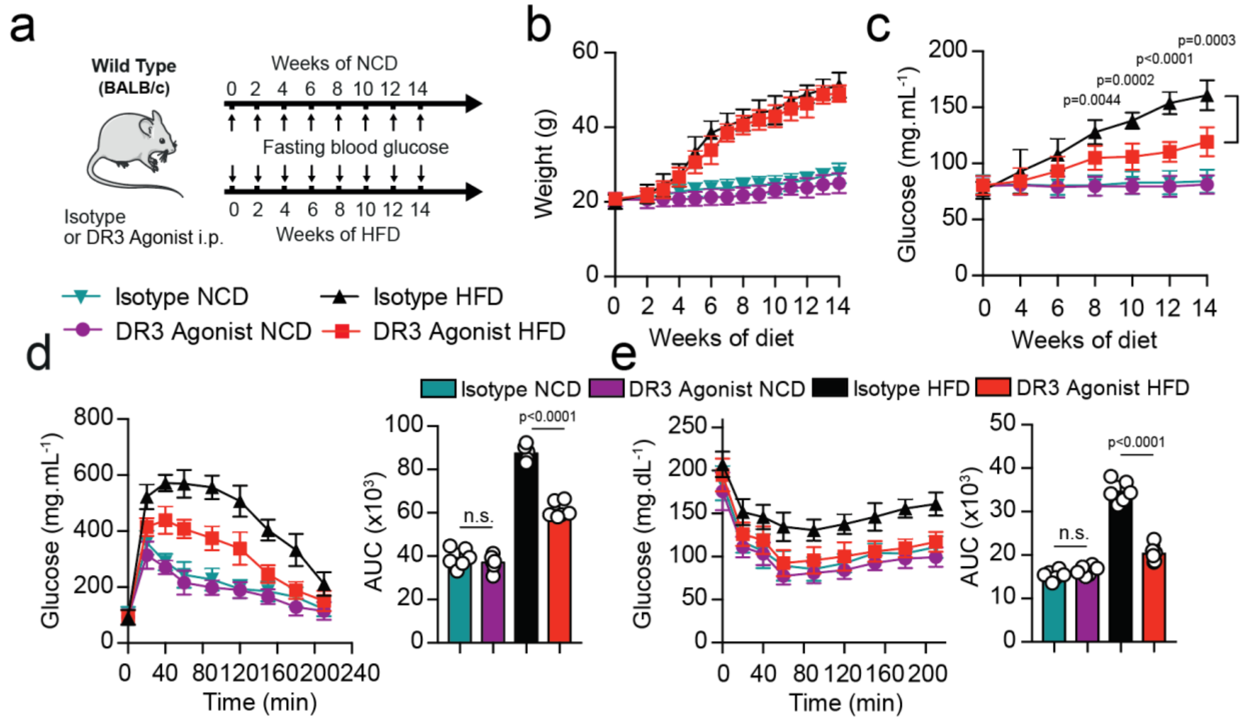

DR3 engagement protects from onset of type 2 diabetes in BALB/cByJ mice. (a) As shown in the timeline, a cohort of BALB/cByJ mice were fed a normal chow diet (NCD) or a high fat diet (HFD). The mice were treated with either DR3 agonist (1mg/mouse) or isotype control via intraperitoneal injections every four days, n=6 mice. **(b)** The total weight was measured every week, and **(c)** the fasting blood glucose levels were measured once every two weeks for a period of 14 weeks. **(d)** Glucose tolerance test, and **(e)** insulin tolerance test were performed after 14 weeks. The corresponding area under the curve (AUC) was calculated for each cohort. Error bars are the mean ± SD. Statistical analysis, one-way ANOVA (b and c), two-way ANOVA (d and e); n.s.: p-value of less than 0.05 was considered as non-significant. Mouse image provided with permission from Servier Medical Art.

Supplementary Figure 2

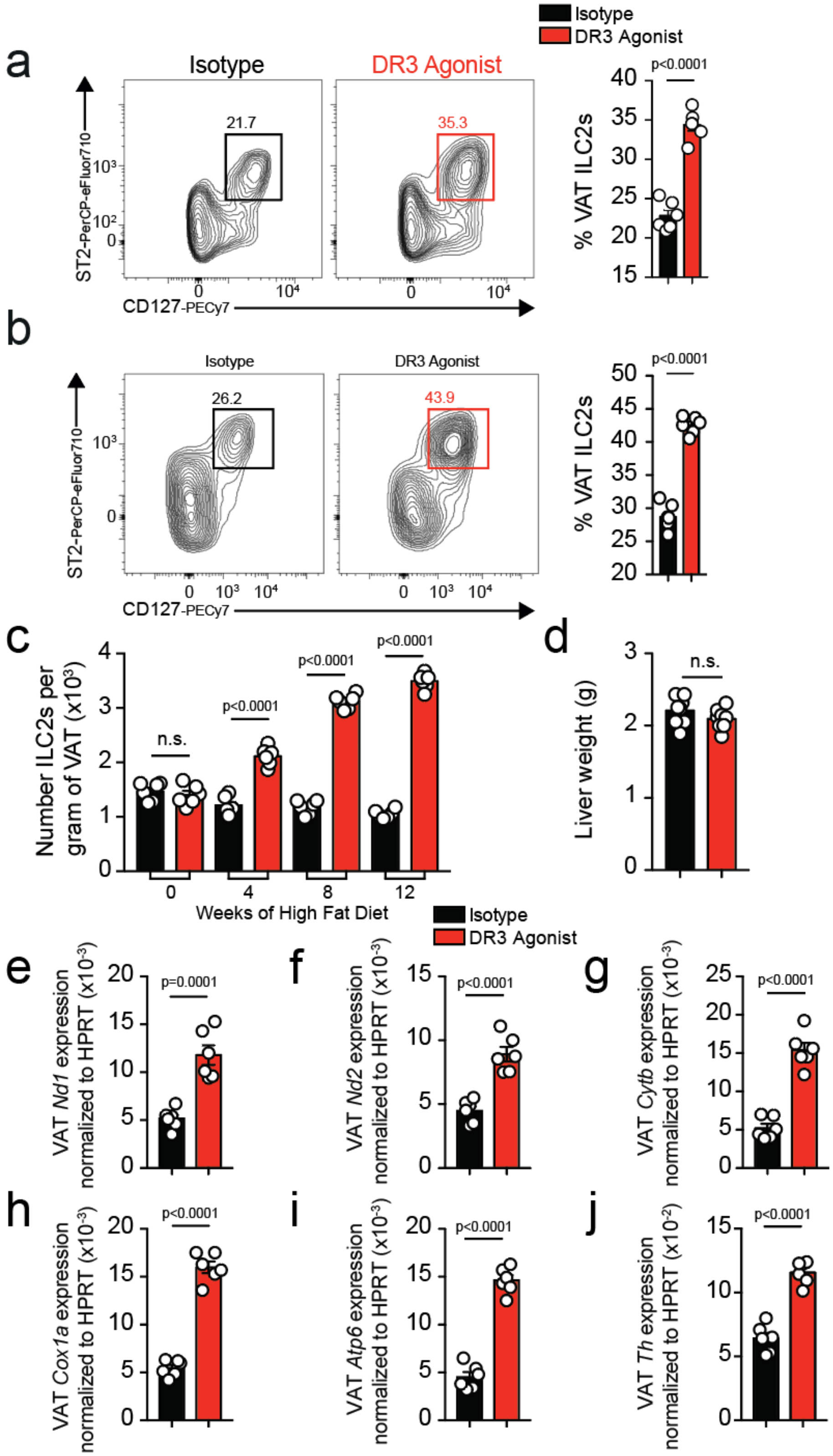

DR3 agonistic treatment augments VAT ILC2s and improves VAT metabolic rate. A cohort of C57/BL6J mice were fed a high fat diet (HFD) and treated with either DR3 agonist (1mg/mouse) or isotype control via intraperitoneal injections every four days, n=6 mice. **(a)** The percentage of VAT ILC2s after 14 weeks of HFD. Similarly, a cohort of *Rag2*-/- were placed on a HFD and treated with DR3 agonist (1mg/mouse) or isotype control once every four days, n=6 mice. The percentage **(b)** and number of VAT ILC2s **(c)** after 14 weeks of HFD. **(d)** Weight of liver. **(e-j)** Transcripts levels of tyrosine hydroxylase (*Th*) and respiratory chain complexes genes (*Nd1*, *Nd2*, *Cytb*, *Cox1a*, and *Atp6*). Error bars are the mean ± SD. Statistical analysis, two-tailed student's t-test; n.s.: p-value of less than 0.05 was considered as non-significant.

Supplementary Figure 3

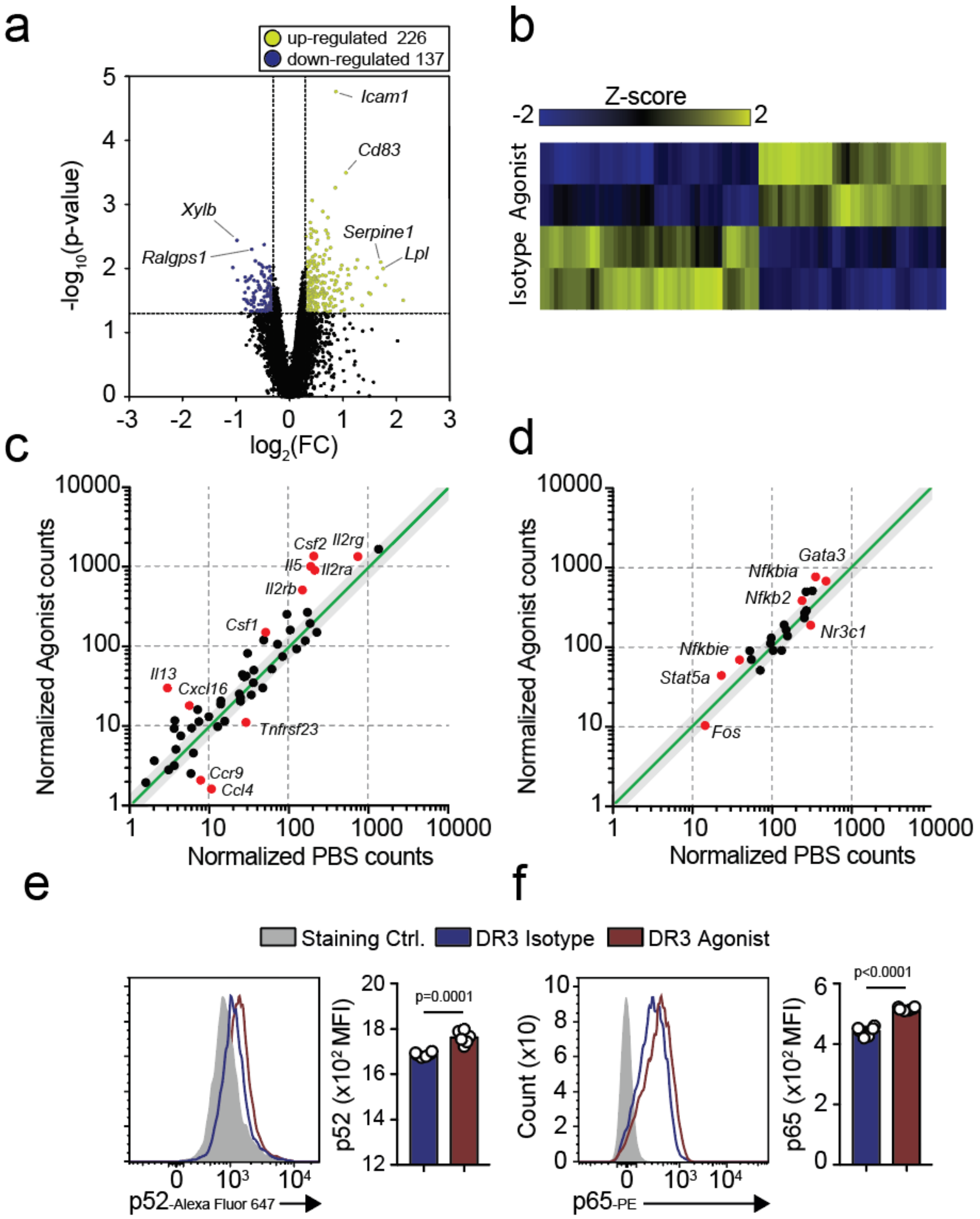

DR3 signaling induces canonical and non-canonical NF-κB pathway in naïve ILC2s. Naïve VAT-derived murine ILC2s (nILC2s) were cultured in presence of recombinant mouse (rm) IL-2, rmIL-7 and stimulated with DR3 agonist (5 µg/mL) or isotype control for 24 hours. Total RNA was

isolated and sequenced. **(a)** Volcano plot comparison representing whole transcriptome gene expression of sorted WT ILC2s treated with either isotype control or DR3 agonist (5µg/mL) for 24 hours in vitro. Differentially expressed genes (described as statistically significant adjusted p-value<0.05) with changes of at least 1.5-fold change (FC) are shown in yellow (upregulated) and blue (downregulated). Relevant differentially expressed genes are identified. **(b)** Heat plot of all differentially expressed genes. Selected cytokine and cytokine receptor genes **(c)** and transcription factors **(d)** plotted as the normalized counts in isotype-treated cohort compared to DR3 stimulated cohort. Notable ILC2 related genes are labeled and highlighted in red. Gray area represents region of 1.5-fold change in gene expression. Representative expression of NF-κB p52 **(e)** and NF-κB p65 **(f)** in isolated ILC2s from naïve mice and cultured in vitro for 24 hours with DR3 agonist (red) or isotype control (blue), n=6 mice. The staining FMO control is shown as grey. The corresponding quantification are presented as Mean Fluorescence Intensity (MFI), and the error bars denote the mean ± SD. Statistical analysis, two-tailed student's t-test (e and f).

Supplementary Figure 4

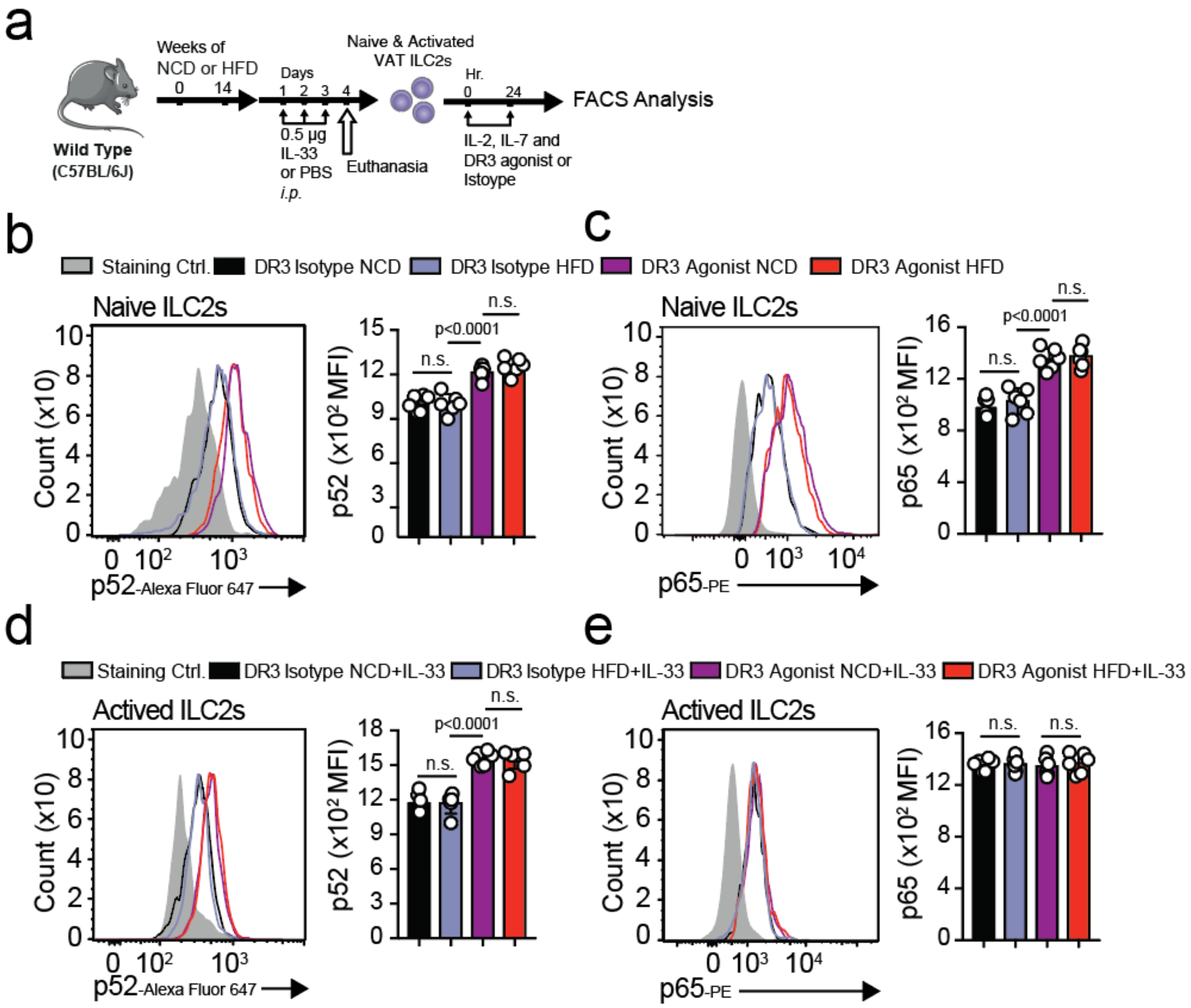

DR3-dependent NF-κB signaling is not impaired on VAT derived ILC2s from obese mice. **(a)** A cohort of C57BL/6J mice were fed a normal chow diet (NCD) or a high fat diet (HFD) for 14 weeks, as shown in the timeline, n=6 mice. The mice were subsequently challenged with recombinant mouse (rm) IL-33 (0.5 µg in 50 µL) or PBS intraperitoneally for three days. The mice were euthanized on fourth day and the visceral adipose tissue (VAT) was isolated. Naïve and activated VAT ILC2s were sorted and freshly cultured in presence of recombinant mouse (rm) IL-2, rmIL-7, and stimulated with DR3 agonist (5 µg/mL) or isotype control *ex vivo* for 24 hours. Representative expression of NF-κB p52 and NF-κB p65 in naïve **(b-c)** and activated **(d-e)** VAT

ILC2s. The staining FMO control is shown as grey. Error bars are the mean ± SD. Statistical analysis, one-way ANOVA; n.s.: p-value of less than 0.05 was considered as non-significant. Mouse image provided with permission from Servier Medical Art.
